# When and how do ‘effective’ interventions need to be adapted and/or re-evaluated in new contexts? The need for guidance

**DOI:** 10.1136/jech-2018-210840

**Published:** 2019-02-20

**Authors:** Rhiannon E Evans, Peter Craig, Pat Hoddinott, Hannah Littlecott, Laurence Moore, Simon Murphy, Alicia O'Cathain, Lisa Pfadenhauer, Eva Rehfuess, Jeremy Segrott, Graham Moore

**Affiliations:** 1 DECIPHer, School of Social Sciences, Cardiff University, Cardiff, UK; 2 MRC/CSO Social and Public Health Sciences Unit, University of Glasgow, Glasgow, UK; 3 Nursing, Midwifery and Allied Health Professions Research Unit (NMAHP RU), University of Stirling, Stirling, UK; 4 Medical Care Research Unit, School of Health and Related Research (SHARR), University of Sheffield, Sheffield, UK; 5 Institute for Medical Informatics, Biometry and Epidemiology, Pettenkofer School of Public Health, LMU Munich, Munich, Germany; 6 DECIPHer, Centre for Trials Research (CTU), Cardiff University, Cardiff, UK

**Keywords:** adaptation, intervention, evaluation, context, population health

Rational models of evidence-informed policy have historically centred on an assumption that it is possible to identify ‘effective’ interventions, before recommending wider implementation. However, for population health interventions (which we define as inclusive of public health and health services), transferability to new contexts is often uncertain.^1^ Some interventions have demonstrated limited effectiveness, or even harm, when used elsewhere. For example, antenatal corticosteroids have reduced neonatal mortality among pregnant women at risk of preterm birth in high-income countries, but increased mortality and maternal infection in low-income and middle-income countries.^2^

While definitions are contested, context can be defined as a set of active and unique characteristics and circumstances that interact with, modify, facilitate or constrain intervention delivery and effects. It includes geographical, epidemiological, sociocultural, socioeconomic, ethical, legal and political determinants.^3^ The argument that every context is unique and interventions cannot translate across them should not be overstated however. Some parenting interventions, for instance, have not successfully transferred, while others have been highly resilient to contextual variation, with minimal difference in effects between ‘home-grown’ and transported approaches.^4^

There is increased recognition then of the need to consider context when making decisions about the transferability of evidence. Population health interventions are increasingly conceived as ‘events in systems’, which aim to modify aspect(s) of a pre-existing context, altering conditions that sustain suboptimal population health outcomes. Effects therefore may be shaped as much by changes to or displacement of prior features of the context, as by properties of the new intervention. Implementation may differ in a new context due to the complexity of the intervention components or ambiguity over its mechanisms, inhibiting high fidelity. Implementation failure may ensue if an intervention conflicts with entrenched cultural norms or requires resources that are not available. Even where implemented as intended, variation in effects may arise from differences in system starting points, with targeted outcomes driven and sustained by different underlying mechanisms.

Assuming that a history of what has worked elsewhere can guarantee future success of similar action in a new time and place is therefore problematic. Equally, assuming effective approaches cannot be transported across contexts is contentious. A systematic understanding of the extant evidence base is important. Large and expensive re-evaluations may not be warranted if an intervention is acceptable and feasible in the new context, and there is robust evidence that the mechanisms targeted by the intervention are relevant. Moreover, there is a need to remain critical about whether purported failures to replicate effects genuinely reflect non-transferability, or are the consequence of the methodologies used in the evaluation in either the original or new context. For example, methods may have become more rigorous as the field develops or the evaluation moves from being conducted by intervention developers to independent investigators.^5^ When transferring ‘evidence-informed’ interventions to a new context, challenging decisions need to be made regarding (1) the need for adaptations to ensure that the intervention can be integrated into a new system, and (2) the level of uncertainty in the transferability of their effect and type of re-evaluation required.

## What does ‘adaptation’ mean?

Although there has been limited conceptual development about what constitutes intervention adaptation, Stirman *et al* 6 broadly describe two domains. First are content modifications, which amend core intervention components. These may be surface-level (eg, enhancing cognitive understanding by amending language) or deep-level (eg, improving cultural fit by responding to the normative value system). Second are contextual modifications, which do not alter content. They may include adaptation to delivery strategies, agents, settings or target populations. The scope for adaptations will likely vary significantly across interventions; highly standardised, licensed interventions may not permit adaptations without permission from the developers. In other instances, interventions include a flexible set of processes that allow the form of components to change so long as the underlying functionality is maintained.

There are numerous examples of adapted interventions, which often include but are not limited to (1) *geographical*: interventions transferred across countries (eg, HIV interventions from the USA to Uganda, which are shown to be as effective in increasing condom use and decreasing number of sexual partners)^7^; and (2) *sociocultural*: interventions replicated within the same geographical context with modifications for population subgroups (eg, mental health interventions for indigenous youth, which have demonstrated the need for implementation models responsive to the local community context of childcare services).^8^

## The need for guidance on adapting population health interventions

In recent years, there has been an emergence of editorials and case studies on how to adapt population health interventions to new contexts. There is, however, no current overarching guidance. As such, current decision-making may be undertaken on an ad-hoc basis. Furthermore, there is a lack of consideration of the importance of describing context, with limited use of frameworks and methods for mapping system-level characteristics.^3 9^ Given the opaque manner in which decisions regarding adaptation are made, it is difficult to disentangle whether replication failures (or successes) in new contexts predominantly stem from intervention fit with the new context, implementation failure, adaptations that violate or enhance the intervention theory, or differences in the methodologies used to assess the transferability of effects. For example, an adapted version of the US-based Strengthening Families Program demonstrated no effect when introduced to Sweden.^10^ While attributed to differences between contexts, commentators argued that local adaptations had gone so far as to violate the intervention’s causal logic.^11^ New guidance is required to respond to these complexities and provide comprehensive and systematic decision-making tools.

## Developing new guidance for the adaptation of complex population health interventions for new contexts

The Medical Research Council (MRC)-National Institute for Health Research (NIHR) Methodology Research Programme in the UK is currently supporting the development of guidance on the adaptation of evidence-informed population health interventions for new contexts. The authors of the present editorial are leading guidance development. Guidance will be developed through inter-related work packages, comprising (1) a systematic review of existing recommendations and a scoping review of interventions implemented and/or re-evaluated in new contexts; (2) qualitative interviews with research, policy and practice stakeholders; and (3) an international Delphi exercise to identify areas of consensus and key theoretical, methodological or substantive uncertainties that can inform the research agenda moving forward. This new guidance will support researchers, policymakers and practitioners in critically evaluating assertions that effects may or may not be contextually contingent, and how to draw on rigorous evidence to establish if adaptation is warranted. It will also support decision-making about the nature and extent of adaptations to be undertaken, how to describe the processes for undertaking adaptations, and the level of re-evaluation required to address uncertainties regarding transferability.

Provision of criteria for assessing the appropriateness of adaptations will assist research funders, journal editors and peer reviewers in resourcing and disseminating the highest quality research. Uptake of guidance across stakeholders will help to overcome the current ad-hoc approach to practice within intervention adaptation. The guidance will build on existing MRC guidance, including guidance for developing and evaluating complex interventions^12^ and process evaluation.^13^ It will complement guidance currently in progress by some of the authors on intervention development (INDEX Study; IdentifyiNg and assessing different approaches to DEveloping compleX interventions), feasibility and pilot studies (GUEST Study; GUidance for Exploratory STudies of complexpublic health interventions), and the role of context within intervention development, evaluation and implementation.
